# The human ehrlichioses in the United States.

**DOI:** 10.3201/eid0505.990504

**Published:** 1999

**Authors:** J H McQuiston, C D Paddock, R C Holman, J E Childs

**Affiliations:** Centers for Disease Control and Prevention, Atlanta, Georgia 30333, USA.

## Abstract

The emerging tick-borne zoonoses human monocytic ehrlichiosis (HME) and human granulocytic ehrlichiosis (HGE) are under reported in the United States. From 1986 through 1997, 1,223 cases (742 HME, 449 HGE, and 32 not ascribed to a specific ehrlichial agent) were reported by state health departments. HME was most commonly reported from southeastern and southcentral states, while HGE was most often reported from northeastern and upper midwestern states. The annual number of reported cases increased sharply, from 69 in 1994 to 364 in 1997, coincident with an increase in the number of states making these conditions notifiable. From 1986 through 1997, 827 probable and confirmed cases were diagnosed by serologic testing at the Centers for Disease Control and Prevention, although how many of these cases were also reported by states is not known. Improved national surveillance would provide a better assessment of the public health importance of ehrlichiosis.


### The Human Ehrlichioses in the United States

**Jennifer H. McQuiston, Christopher D. Paddock, Robert C. Holman, and James E. Childs**
Centers for Disease Control and Prevention, Atlanta, Georgia, USA

The emerging tick-borne zoonoses human monocytic ehrlichiosis (HME) and human granulocytic ehrlichiosis (HGE) are underreported in the United States. From 1986 through 1997, 1,223 cases (742 HME, 449 HGE, and 32 not ascribed to a specific ehrlichial agent) were reported by state health departments. HME was most commonly reported from southeastern and southcentral states, while HGE was most often reported from northeastern and upper midwestern states. The annual number of reported cases increased sharply, from 69 in 1994 to 364 in 1997, coincident with an increase in the number of states making these conditions notifiable. From 1986 through 1997, 827 probable and confirmed cases were diagnosed by serologic testing at the Centers for Disease Control and Prevention, although how many of these cases were also reported by states is not known. Improved national surveillance would provide a better assessment of the public health importance of ehrlichiosis.

First recognized in the United States in 1986, the human ehrlichioses are considered emerging zoonotic diseases. Two etiologically and epidemiologically distinct forms of illness are recognized: human monocytic ehrlichiosis (HME), caused by *Ehrlichia chaffeensis* (1), and human granulocytic ehrlichiosis (HGE), caused by an agent similar or identical to the veterinary pathogens *E. equi* and *E. phagocytophila* (2). A third species, *E. ewingii*, can also cause human illness (3). The bacteria that cause ehrlichiosis are transmitted to humans through the bite of infected ticks, which acquire the agents after feeding on infected animal reservoirs.

During infection, ehrlichiae form distinctive membrane-bound, intracytoplasmic bacterial aggregates (morulae) in white blood cells. HME is characterized by morulae in monocytes, HGE by morulae in granulocytes. Clinically, HME and HGE are nearly indistinguishable and are characterized by one or more of the following symptoms: fever, headache, myalgia, thrombocytopenia, leukopenia, and elevated liver enzyme levels (4-8). A rash occurs in approximately one third of patients with HME (8) but is less common in patients with HGE (4,9). Most cases of ehrlichiosis are characterized by mild illness. However, complications such as adult respiratory distress syndrome, renal failure, neurologic disorders, and disseminated intravascular coagulation can occur (6,10). Case-fatality ratios are as high as 5% for HME and 10% for HGE (10), although more serious cases are probably overrepresented in these estimates. Other studies have reported case-fatality ratios of <5% for these diseases (4,7).

HME and HGE are most often diagnosed by indirect immunofluorescence assay (IFA), although polymerase chain reaction (PCR) assays are increasingly used (11). A confirmed case is defined as a fourfold change in antibody titer by IFA in acute- and convalescent-phase serum samples, PCR amplification of ehrlichial DNA from a clinical sample, or detection of intraleukocytic morulae and a single IFA titer of $\geq 64$. A probable case is defined as a single IFA titer of $\geq 64$ or the presence of morulae within infected leukocytes. Laboratory data are only used to support clinical suspicion; the designation of a confirmed or probable case of ehrlichiosis is interpreted in the context of compatible illness (11).

The public health importance of the ehrlichioses has not been well defined, largely because these diseases are newly recognized. Because ehrlichiae are present in blood, concerns have been raised about the risk for perinatal and blood-transfusion transmission

Address for correspondence: J.E. Childs, Centers for Disease Control and Prevention, Mail Stop G13, 1600 Clifton Road, Atlanta, GA 30333, USA; fax: 404-639-2778; e-mail: jfc5@cdc.gov.

(12,13). Ehrlichiae are susceptible to tetracyclines, so rapid and effective treatment is possible (8). However, the nonspecific signs and symptoms of these diseases may interfere with timely clinical diagnosis. Ehrlichial infections can be life-threatening. Raising disease awareness and educating physicians and the public about clinical manifestations and proper treatment are indicated.

A national ehrlichiosis surveillance program does not exist, so national incidence rates have not been determined because of wide variability in state surveillance activities. The Council of State and Territorial Epidemiologists recommended that human ehrlichiosis be made nationally notifiable in 1998, but many states do not have a system for surveillance and do not test for ehrlichiosis in state diagnostic laboratories. We summarize the scope of state-supported surveillance efforts and present data on ehrlichiosis cases reported to state health departments from 1986 through 1997. In addition, we include data on ehrlichiosis cases diagnosed by serologic testing at the Centers for Disease Control and Prevention (CDC).

### Reported Ehrlichiosis Cases in the United States

From 1986 through 1997, 1,223 ehrlichiosis cases were reported by 30 state health departments in the United States. Data were reported from 19 states that considered ehrlichiosis notifiable as of August 1998, five that routinely collected information on cases, and six that occasionally received reports of ehrlichiosis cases (Appendix I) (14-17). For states where ehrlichiosis was not notifiable, the designation routine reporting versus occasional reporting was based on the completeness of data provided. Because some states did not differentiate between probable and confirmed cases in their records, both categories were considered cases for the purposes of this report. Of the 1,223 reported ehrlichiosis cases, 742 (60.7%) were categorized as HME, 449 (36.7%) as HGE, and 32 (2.6%) as not ascribed to a specific ehrlichial agent. Using data from 20 states that reported information on deaths, we found case-fatality ratios of 2.7% (8 of 299) for HME and 0.7% (3 of 448) for HGE.

### HME and HGE Incidence

Data provided through 1997 were used to calculate state-specific average annual incidence rates for 16 of the 19 states that considered ehrlichiosis notifiable and the five states that routinely collected surveillance data (Table). Although Missouri, South Carolina, and Tennessee considered ehrlichiosis notifiable, average annual incidence rates could not be calculated because these states did not differentiate between HME and HGE. Average annual incidence per one million population was calculated by dividing the number of reported cases by the number of years a state collected data (Table). When possible, average annual incidence by county was determined for HME and HGE (Figures 1-2) (15,17).

Table. Average annual ehrlichiosis incidence (per one million population) for reporting states[a] on the basis of 1995 census data (18)

| State | Incidence: Human monocytic ehrlichiosis | Incidence: Human granulocytic ehrlichiosis |
|---|---|---|
| Arkansas | 5.53 | 0 |
| Arizona | 0.12 | 0 |
| California | 0.02 | 0.03 |
| Connecticut | 0.92 | 15.90 |
| Florida | 0.74 | 0 |
| Illinois | 0.11 | 0.03 |
| Indiana | 0.91 | 0 |
| Kentucky | 0.40 | 0 |
| Maine | 0 | 0 |
| Minnesota | 0.22 | 3.90 |
| Missouri | 3.05 | 0 |
| North Carolina | 4.72 | 0.05 |
| New Hampshire | 0 | 0 |
| New Jersey | 1.47 | 0.17 |
| New York | 0.38 | 2.68 |
| Oklahoma | 2.90 | 0 |
| Pennsylvania | 0.01 | 0.03 |
| Rhode Island | 0 | 0.67 |
| Texas | 0.20 | 0 |
| Virginia | 0.68 | 0 |
| Wisconsin | 0 | 8.79 |

[a]Includes states that consider ehrlichiosis notifiable, as well as five states where data are routinely collected. Michigan, South Carolina, and Tennessee did not differentiate between cases of human monocytic ehrlichiosis and human granulocytic ehrlichiosis and are not included in this table.

Most HME cases were reported from the southeastern and southcentral areas of the United States (Table, Figure 1). The highest reported average annual incidence rates of HME were in Arkansas (5.53 per million), North Carolina (4.72 per million), Missouri (3.05 per million), and Oklahoma (2.90 per million). In contrast, the highest reported average annual incidence rates

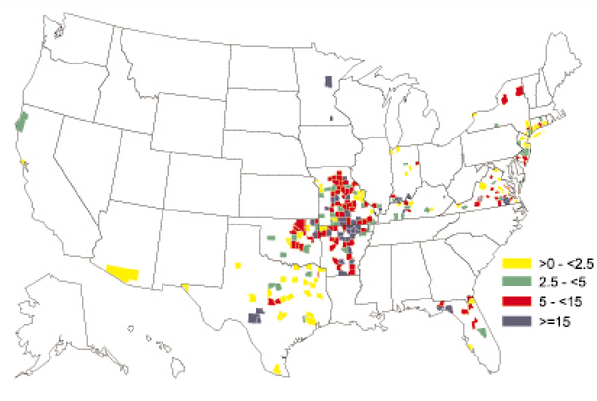

Figure 1. Average annual incidence of reported human monocytic ehrlichiosis (HME) by county, using 1995 population census data (29). Includes states that consider ehrlichiosis notifiable, as well as states that routinely collect information on ehrlichiosis cases. Michigan, South Carolina, and Tennessee are not included because cases of HME and human granulocytic ehrlichiosis were not distinguished by the state health departments. County-specific incidence could not be calculated for North Carolina or Pennsylvania because county of occurrence was not provided by the state health departments.

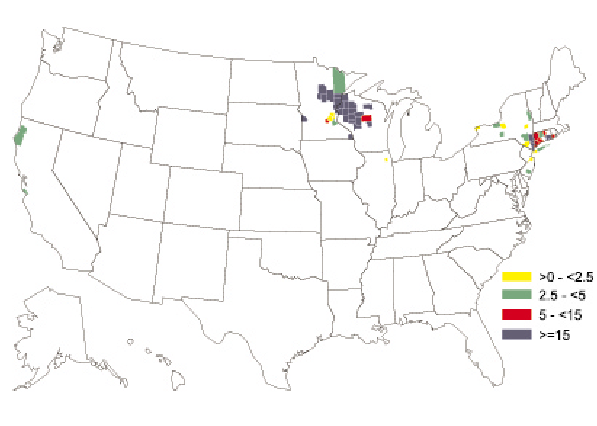

Figure 2. Average annual incidence of reported human granulocytic ehrlichiosis (HGE) by county, using 1995 population census data (29). Includes states that consider ehrlichiosis notifiable, as well as states that routinely collect information on ehrlichiosis cases. Michigan, South Carolina, and Tennessee are not included because cases of human monocytic ehrlichiosis and HGE were not distinguished by the state health departments. County-specific incidence could not be calculated for North Carolina or Pennsylvania because county of occurrence was not provided by the state health departments.

of HGE were in the northeastern and upper midwestern areas of the United States—Connecticut (15.90 per million), Wisconsin (8.79 per million), Minnesota (3.90 per million), and New York (2.68 per million) (Figure 2). The county reporting the highest average annual incidence of HME was Searcy, Arkansas (64.80 per million), and the county with the highest annual incidence of HGE was Jackson, Wisconsin (521.68 per million).

These incidence rates follow the expected geographic distribution of tick vectors for each type of ehrlichiosis. *E. chaffeensis* is primarily transmitted by the lone star tick *(Amblyomma americanum),* which is common in the southeastern United States (19). The black-legged tick *(Ixodes scapularis)* transmits the causative agent of HGE in the northeastern United States (20,21) and the western black-legged tick *(I. pacificus)* in the western coastal United States (22).

### Reporting Trends

The annual number of ehrlichiosis cases reported by the state health departments was calculated with data from 18 states that considered ehrlichiosis notifiable as of August 1998 (yearly summaries were not available for Missouri) and the five additional states that routinely collected information on ehrlichiosis cases (Figure 3). The annual number of reported

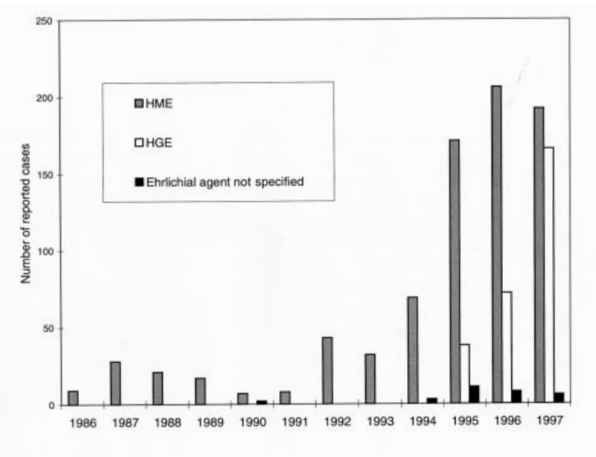

Figure 3. Reported cases of human monocytic ehrlichiosis (HME) and human granulocytic ehrlichiosis (HGE) in the United States, 1986-1997 (includes cases from states that consider ehrlichiosis notifiable, as well as states that routinely collect information). Because yearly summaries of reported cases were not available for Missouri, data from this state are not included. The number of states where ehrlichiosis was notifiable increased from 7 in 1994 to 17 in 1997.

ehrlichiosis cases increased sharply, from 69 in 1994 to 364 in 1997. This increase may be explained by the addition of ehrlichiosis as a notifiable disease in 10 states during this same 4-year interval, the discovery of HGE in 1994, increased availability of diagnostic tests, and increased awareness of ehrlichiosis.

### Ehrlichioses Cases Diagnosed at CDC

At CDC, antigen from *E. chaffeensis,* Arkansas strain, is used to diagnose HME by IFA. Before *E. chaffeensis* was isolated in 1991, *E. canis* was used as a surrogate antigen (23). During 1995 to 1996, antigen from *E. equi* obtained from infected horse neutrophils was used, but cases submitted to CDC after 1996 were diagnosed by IFA using cell culture–derived antigen from the HGE agent (24). Antibody from patients with ehrlichial infection may cross-react with both *E. chaffeensis* and the HGE agent (24,25). For patients with significant antibody titers to both *Ehrlichia* species, the causative agent is assumed to be the one with a fourfold or greater change in antibody titer between paired serum samples. If both agents show a fourfold difference, the one with the highest titer is considered the causative agent. If neither shows a fourfold difference, the causative agent is usually not ascribed to a specific ehrlichial species (25).

Of 827 probable and confirmed ehrlichiosis cases diagnosed by IFA from serum or plasma specimens submitted to CDC through the end of 1997, 754 were HME, 44 were HGE, and 29 could not be differentiated because of antibody cross-reactivity. The geographic distribution was widespread (Figures 4, 5), and cases of ehrlichiosis were diagnosed from every state except North Dakota and South Dakota (Appendix 2). Imported disease acquired by travel to disease-endemic areas may explain cases reported from states without the recognized tick vectors, including Hawaii and Alaska. Because information about clinical manifestations was not always provided with specimens, whether all cases had compatible clinical illness is unknown. Of 754 HME cases, 423 (56.1%) were classified as probable and 331 (43.9%) as confirmed on the basis of serologic criteria established by CSTE and CDC (11). In contrast, of 44 HGE cases, 39 (88.6%) were classified as probable and 5 (11.4%) as confirmed.

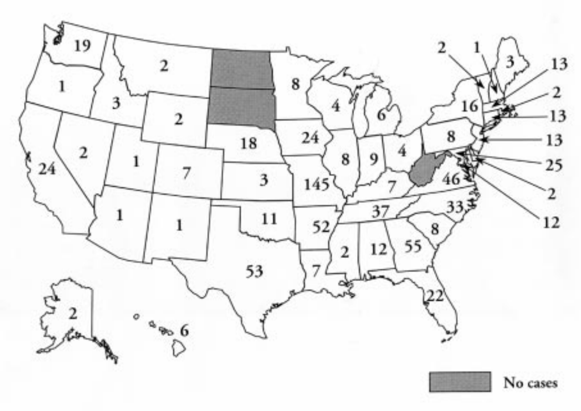

Figure 4. Human monocytic ehrlichiosis cases diagnosed by indirect immunofluorescence assay (IFA), Centers for Disease Control and Prevention, 1986 to 1997.

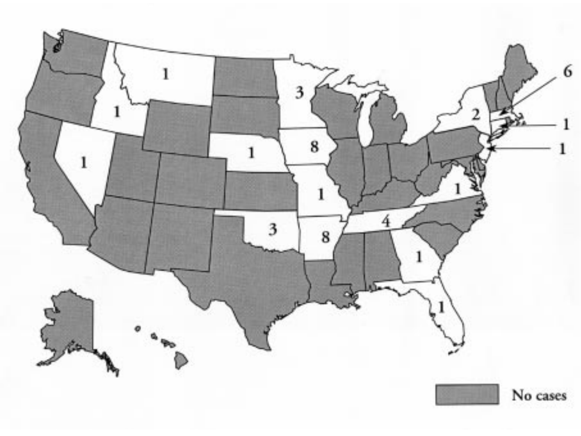

Figure 5. Human granulocytic ehrlichiosis cases diagnosed by indirect immunofluorescence assay (IFA), Centers for Disease Control and Prevention, 1995 to 1997.

### Conclusions

Although a few state health departments have published information on local ehrlichiosis surveillance (14-17,26-28), comprehensive national surveillance data had not been collected until this review. This review further defines the public health problem posed by the ehrlichioses in the United States. These diseases have incidence rates comparable with or exceeding those of Rocky Mountain spotted fever in some states (29).

These state-reported data have several limitations. State health departments provided information on ehrlichiosis cases in different ways. For example, some states provided only data compiled after ehrlichiosis became notifiable, while others provided information as far back as data were available. The ehrlichiosis cases in this article represent a compilation of existing (albeit incomplete) surveillance datasets and probably underestimate the true prevalence of the disease in the United States. Moreover, the accuracy of HME and HGE case-fatality ratios presented here is uncertain. The number of deaths may be underreported because diagnosis of ehrlichiosis requires laboratory confirmation. However, serious or complicated cases, more likely to end in death, are more likely to be investigated and reported to state health departments. The case-fatality ratios described in this article are compatible with findings from other studies (4,7). Finally, the state-reported data include some cases from areas where ehrlichiosis is not commonly diagnosed. For example, a single case of HME was reported from Arizona, although the recognized distribution of the lone star tick does not include this state. Ehrlichiosis cases are usually reported from the patient's county and state of residence at the time of diagnosis; however, ehrlichiosis may be acquired during travel to an area with *Ehrlichia*-infected ticks. Imported cases of ehrlichiosis in states where the disease is not common or tick vectors are absent underscores the need to consider this diagnosis even in areas of low risk.

Diagnostic serologic testing has been offered at CDC since 1986 for HME, and since 1995 for HGE. Records show that from 1986 through 1997 more than 800 ehrlichiosis cases were diagnosed from 48 states. This finding contrasts sharply with state-reported surveillance data, which identified specific geographic regions where ehrlichiosis was most likely to occur.

The number of cases diagnosed at CDC from each state may not accurately reflect expected regional incidence patterns; for example, states with public health laboratories that offer in-house diagnostic tests or states that frequently use commercial laboratories may be less likely to submit samples to CDC for testing. Some cases of ehrlichiosis diagnosed at CDC may also have been reported by state surveillance systems; these reporting systems cannot be regarded as mutually exclusive. The numbers of serologically diagnosed cases of ehrlichiosis reported here may differ from numbers published in other CDC reports because other reports include samples obtained for specific studies (7), whereas most of the cases in this report were submitted for routine diagnostic tests.

As of August 1998, only 19 states considered ehrlichiosis notifiable, and fewer than one fourth of state health departments offered in-house diagnostic assays for HME or HGE. Average annual incidence rates, an important indicator of disease prevalence, could be calculated for only 21 states. These data underscore the need for better nationwide surveillance of ehrlichiosis.

### Acknowledgments

We thank the state health departments for sharing these data and gratefully acknowledge the contributions of Bob England, Thomas McChesney, Dennis Berry, Thomas Tsang, Mark Starr, David Schnurr, John Poppy, Jim Meek, Cathy Rebmann, Lisa Conti, Valerie Mock, Jeff Chapman, Mike Loeffelholz, Carl Langkop, Robert Teclaw, Pam Judson, Mike Auslander, Louise McFarland, Mike McGuill, David Blythe, Linda Traviti, Jeff Beckett, Mary Grace Stobierski, Sheril Arndt, Dave Neitzel, Mary Currier, Melissa Johnson, Faye Sorhage, Lorna Lynch, Paul Edistat, Philip Kurpiel, Susan Wong, Todd McPherson, Leslie Wolf, Mike Crutcher, Maria Moll, Ted Donnelly, Ted LeBlanc, Steve McLaughlin, Robert Taylor, Jerry Hindman, Julie Rawlings, Farah Babakhani, Les Branch, John Kobayashi, Jim Kazmierczak, and Ed Belongia.

We thank Joseph Singleton for help in collecting data on confirmed and probable ehrlichiosis cases diagnosed at CDC.

Dr. McQuiston, a veterinarian, is serving as an Officer in the Epidemic Intelligence Service, Centers for Disease Control and Prevention. Her research focuses on the epidemiologic investigation of several zoonotic pathogens, including rabies virus, ehrlichioses, and *Rickettsia rickettsii*.

### References

1. Anderson BE, Dawson JE, Jones DC, Wilson KH. *Ehrlichia chaffeensis,* a new species associated with human ehrlichiosis. J Clin Microbiol 1991;29:2838-42.
2. Chen S, Dumler JS, Bakken JS, Walker DH. Identification of a granulocytotropic *Ehrlichia* species as the etiologic agent of human disease. J Clin Microbiol 1994;32:589-95.
3. Hmiel SP, Buller R, Arens M, Gaudreault-Keener M, Storch GA. Human infection with *Ehrlichia ewingii*, the agent of Ozark canine granulocytic ehrlichiosis. Proceedings of the First International Conference on Emerging Infectious Diseases; 1998 Mar 8-11; Atlanta, Georgia; Addendum:4. [Abstract].
4. Bakken JS, Krueth J, Wilson-Nordskog C, Tilden RL, Asanovich K, Dumler JS. Clinical and laboratory characteristics of human granulocytic ehrlichiosis. JAMA 1996;275:199-205.
5. Dawson JE, Warner CK, Standaert S, Olson JG. The interface between research and the diagnosis of an emerging tick-borne disease, human ehrlichiosis due to *Ehrlichia chaffeensis.* Arch Intern Med 1996;156:137-42.

6. Eng TR, Harkess JR, Fishbein DB, Dawson JE, Greene CN, Redus MA, et al. Epidemiologic, clinical, and laboratory findings of human ehrlichiosis in the United States, 1988. JAMA 1990;264:2251-8.
7. Fishbein DB, Dawson JE, Robinson LE. Human ehrlichiosis in the United States, 1985 to 1990. Ann Intern Med 1994;120:736-43.
8. Fritz CL, Glaser CA. Ehrlichiosis. Infect Dis Clin North Am 1998;12:123-36.
9. Aguero-Rosenfeld ME, Horowitz HW, Wormser GP, McKenna DF, Nowakowski J, Munoz J, et al. Human granulocytic ehrlichiosis: a case series from a medical center in New York state. Ann Intern Med 1996;125:904-8.
10. Dumler JS, Bakken JS. Ehrlichial diseases of humans: emerging tick-borne infections. Clin Infect Dis 1995;20:1102-10.
11. Centers for Diseases Control and Prevention. Case definitions for infectious conditions under public health surveillance. MMWR Morb Mortal Wkly Rep 1997;46:46-7.
12. Arguin PM, Singleton J, Rotz LD, Marston E, Treadwell TA, Slater K, et al. An investigation into the possibility of transmission of tick-borne pathogens via blood transfusion. Transfusion. In press 1999.
13. Horowitz HW, Kilchevsky E, Haber S, Aguero-Rosenfeld M, Kranwinkel R, James EK, et al. Perinatal transmission of the agent of human granulocytic ehrlichiosis. N Engl J Med 1998;339:375-8.
14. Belongia E, Reed K, Mitchell P, Chyou P, Persing D, Finkel M, et al. Active surveillance for human granulocytic ehrlichiosis (HGE) in Northwestern Wisconsin: first year results. Proceedings of the First International Conference on Emerging Infectious Diseases; 1998 Mar 8-11; Atlanta, Georgia; p. 106 [abstract].
15. Hardin LE, Satalowich FT. Tick-borne disease summary—1997. Missouri Epidemiologist; 1998. p. 6-9.
16. Rawlings J. Human ehrlichiosis in Texas. Journal of Spirochetal and Tick-Borne Diseases 1996;3:94-7.
17. Satalowich FT. Tick-borne disease summary—1996. Missouri Epidemiologist; 1997. p. 10-2.
18. Bureau of Census. Intercensual estimates of the population of counties by age, sex, and race: 1995. Washington: The Bureau; 1996.
19. Anderson BE, Sims KG, Olson JG, Childs JE, Piesman JR, Happ CM, et al. *Amblyomma americanum*: a potential vector of human ehrlichiosis. Am J Trop Med Hyg 1993;49:239-44.
20. Magnarelli LA, Stafford KC, Mather TN, Yeh M, Horn KD, Dumler JS. Hemocytic rickettsia-like organisms in ticks: serologic reactivity with antisera to Ehrlichiae and detection of DNA of the agent of human granulocytic ehrlichiosis by PCR. J Clin Microbiol 1995;33:2710-4.
21. Pancholi P, Kolbert CP, Mitchell PD, Reed KD, Dumler JS, Bakken JS, et al. *Ixodes dammini* as a potential vector of human granulocytic ehrlichiosis. J Infect Dis 1995;172:1007-12.
22. Richter PJ Jr, Kimsey RB, Madigan JE, Barlough JE, Dumler JS, Brooks DL. *Ixodes pacificus* (Acari: Ixodidae) as a vector of *Ehrlichia equi* (Rickettsiales: Ehrlichieae). J Med Entomol 1996;33:1-5.
23. Dawson JE, Rikihisa Y, Ewing SA, Fishbein DB. Serologic diagnosis of human ehrlichiosis using two *Ehrlichia canis* isolates. J Infect Dis 1991;163:564-7.
24. Nicholson WL, Comer JA, Sumner JW, Gingrich-Baker C, Coughlin RT, Magnarelli LA, et al. An indirect immunofluorescence assay using a cell culture-derived antigen for detection of antibodies to the agent of human granulocytic ehrlichiosis. J Clin Microbiol 1997;35:1510-6.
25. Comer JA, Nicholson WL, Olson JG, Childs JE. Serologic testing for human granulocytic ehrlichiosis at a national referral center. J Clin Microbiol 1999;37:558-64.
26. Centers for Disease Control and Prevention. Human ehrlichiosis—Maryland, 1994. MMWR Morb Mortal Wkly Rep 1996;45:798-802.
27. Centers for Disease Control and Prevention. Human granulocytic ehrlichiosis—New York, 1995. MMWR Morb Mortal Wkly Rep 1995;44:593-5.
28. Centers for Disease Control and Prevention. Statewide surveillance for ehrlichiosis—Connecticut and New York, 1994-1997. MMWR Morb Mortal Wkly Rep 1998;47:476-80.
29. Dalton MJ, Clarke MJ, Holman RC, Krebs JW, Fishbein DB, Olson JG, et al. National surveillance for Rocky Mountain spotted fever, 1981-1992: epidemiologic summary and evaluation of risk factors for fatal outcome. Am J Trop Med Hyg 1995;52:405-13.

Appendix I: Ehrlichiosis surveillance by state health departments as of August 1998 and total number of cases reported through 1997.

| State | First year reportable | Laboratory Tests offered | Human monocytic ehrlichiosis cases | Human granulocytic ehrlichiosis cases | Ehrlichial agent not specified | Total cases |
|---|---|---|---|---|---|---|
| Alabama | Not reportable | None available | -- | -- | -- | -- |
| Alaska | Not reportable | None available | -- | -- | -- | -- |
| Arizona | 1997 | None available | 1 | 0 | 0 | 1 |
| Arkansas | 1993 | None available | 55 | 0 | 0 | 55 |
| California | 1996 | IFA for both; PCR for both | 2 | 3 | 0 | 5 |
| Colorado | Not reportable[a] | None available | -- | -- | 3 | 3 |
| Connecticut | 1995 | IFA for both; PCR for both | 9 | 156 | 9 | 174 |
| Delaware | Not reportable | None available | -- | -- | -- | -- |
| District of Columbia | Not reportable | None available | -- | -- | -- | -- |
| Florida | 1996 | IFA for HME only | 21 | 0 | 0 | 21 |
| Georgia | Not reportable | None available | -- | -- | -- | -- |
| Hawaii | Not reportable | None available | -- | -- | -- | -- |
| Idaho | Not reportable | None available | -- | -- | -- | -- |
| Illinois | Not reportable[b] | None available | 5 | 1 | 2 | 8 |
| Indiana | Not reportable[b] | IFA for HME only | 21 | 0 | 0 | 21 |
| Iowa | Not reportable | IFA for both; PCR for both | -- | -- | -- | -- |
| Kansas | Not reportable | None available | -- | -- | -- | -- |
| Kentucky | 1989 | None available | 14 | 0 | 0 | 14 |
| Louisiana | Not reportable[a] | None available | -- | -- | 1 | 1 |
| Maine | 1996 | None available | 0 | 0 | 0 | 0 |
| Maryland | Not reportable[a] | IFA for both | 6 | 0 | 1 | 7 |
| Massachusetts | Not reportable[a] | None available | 0 | 5 | 0 | 5 |
| Michigan | 1993 | None available | -- | -- | 2 | 2 |
| Minnesota | 1996 | None available | 2 | 36 | 0 | 38 |
| Mississippi | Not reportable[a] | None available | -- | -- | 1 | 1 |
| Missouri | Reportable, date unknown | None available | 162 | 0 | 0 | 162 |
| Montana | Not reportable | None available | -- | -- | -- | -- |
| Nebraska | Not reportable | None available | -- | -- | -- | -- |
| Nevada | Not reportable | None available | -- | -- | -- | -- |
| New Hampshire | 1996 | None available | 0 | 0 | 0 | 0 |
| New Jersey | 1995 | IFA for both; PCR for both | 35 | 4 | 0 | 39 |
| New Mexico | Not reportable[a] | None available | 1 | 0 | 0 | 1 |
| New York | 1996 | IFA for both; PCR for both | 28 | 195 | 0 | 223 |
| North Carolina | 1998 | IFA for HME only | 204 | 1 | 0 | 205 |
| North Dakota | Not reportable | None available | -- | -- | -- | -- |
| Ohio | Not reportable | None available | -- | -- | -- | -- |
| Oklahoma | Not reportable[b] | None available | 76 | 0 | 0 | 76 |
| Oregon | Not reportable | None available | -- | -- | -- | -- |
| Pennsylvania | 1992 | None available | 1 | 1 | 1 | 3 |
| Rhode Island | 1996 | None available | 0 | 2 | 0 | 2 |
| South Carolina | 1990 | None available | -- | -- | 5 | 5 |
| South Dakota | Not reportable | None available | -- | -- | -- | -- |
| Tennessee | 1996 | IFA for HME only | -- | -- | 7 | 7 |
| Texas | 1996 | IFA for HME only; PCR for both | 45 | 0 | 0 | 45 |
| Utah | Not reportable | None available | -- | -- | -- | -- |
| Vermont | Not reportable | None available | -- | -- | -- | -- |
| Virginia | Not reportable[b] | None available | 54 | 0 | 0 | 54 |
| Washington | Not reportable | None available | -- | -- | -- | -- |
| West Virginia | Not reportable | None available | -- | -- | -- | -- |
| Wisconsin | Not reportable[b] | IFA for both; PCR for HGE only | 0 | 45 | 0 | 45 |
| Wyoming | Not reportable | None available | -- | -- | -- | -- |
| Total | n/a | n/a | 742 | 449 | 32 | 1,223 |

[a]Occasionally received reports of ehrlichiosis cases.
[b]Routinely collected information on ehrlichiosis cases.
HME, human monocytic ehrlichiosis; HGE, human granulocytic ehrlichiosis; -–, not reported by states; IFA, indirect immunofluorescence assay; PCR, polymerase chain reaction; n/a, not applicable.

Appendix II: Probable and confirmed ehrlichiosis cases diagnosed by indirect immunfluorescence assay (IFA), Centers for Disease Control and Prevention, 1986 through 1997.

| | Human monocytic ehrlichiosis | | | Human granulocytic ehrlichiosis | | | Ehrlichial agent not determined[a] | | | Total |
|---|---|---|---|---|---|---|---|---|---|---|
| State | Prob[b] | Conf[c] | Total | Prob[b] | Conf[c] | Total | Prob[b] | Conf[c] | Total | cases |
| Alabama | 8 | 4 | 12 | 0 | 0 | 0 | 0 | 1 | 1 | 13 |
| Alaska | 2 | 0 | 2 | 0 | 0 | 0 | 0 | 0 | 0 | 2 |
| Arizona | 0 | 1 | 1 | 0 | 0 | 0 | 0 | 0 | 0 | 1 |
| Arkansas | 32 | 20 | 52 | 8 | 0 | 8 | 1 | 1 | 2 | 62 |
| California | 15 | 9 | 24 | 0 | 0 | 0 | 0 | 0 | 0 | 24 |
| Colorado | 6 | 1 | 7 | 0 | 0 | 0 | 0 | 0 | 0 | 7 |
| Connecticut | 9 | 4 | 13 | 0 | 1 | 1 | 0 | 0 | 0 | 14 |
| Delaware | 1 | 1 | 2 | 0 | 0 | 0 | 0 | 0 | 0 | 2 |
| District of Columbia | 4 | 8 | 12 | 0 | 0 | 0 | 1 | 0 | 1 | 13 |
| Florida | 15 | 7 | 22 | 0 | 1 | 1 | 2 | 1 | 3 | 26 |
| Georgia | 30 | 25 | 55 | 1 | 0 | 1 | 1 | 0 | 1 | 57 |
| Hawaii | 2 | 4 | 6 | 0 | 0 | 0 | 0 | 0 | 0 | 6 |
| Idaho | 3 | 0 | 3 | 1 | 0 | 1 | 0 | 0 | 0 | 4 |
| Illinois | 3 | 5 | 8 | 0 | 0 | 0 | 0 | 0 | 0 | 8 |
| Indiana | 9 | 0 | 9 | 0 | 0 | 0 | 0 | 0 | 0 | 9 |
| Iowa | 19 | 5 | 24 | 8 | 0 | 8 | 2 | 0 | 2 | 34 |
| Kansas | 1 | 2 | 3 | 0 | 0 | 0 | 0 | 0 | 0 | 3 |
| Kentucky | 5 | 2 | 7 | 0 | 0 | 0 | 0 | 0 | 0 | 7 |
| Louisiana | 5 | 2 | 7 | 0 | 0 | 0 | 0 | 0 | 0 | 7 |
| Maine | 2 | 1 | 3 | 0 | 0 | 0 | 0 | 0 | 0 | 3 |
| Maryland | 18 | 7 | 25 | 0 | 0 | 0 | 1 | 1 | 2 | 27 |
| Massachusetts | 10 | 3 | 13 | 5 | 1 | 6 | 1 | 1 | 2 | 21 |
| Michigan | 4 | 2 | 6 | 0 | 0 | 0 | 0 | 0 | 0 | 6 |
| Minnesota | 5 | 3 | 8 | 0 | 0 | 0 | 0 | 0 | 0 | 8 |
| Mississippi | 0 | 2 | 2 | 0 | 0 | 0 | 0 | 0 | 0 | 2 |
| Missouri | 61 | 84 | 145 | 0 | 1 | 1 | 2 | 2 | 4 | 150 |
| Montana | 2 | 0 | 2 | 1 | 0 | 1 | 0 | 0 | 0 | 3 |
| Nebraska | 17 | 1 | 18 | 1 | 0 | 1 | 0 | 1 | 1 | 20 |
| Nevada | 0 | 1 | 1 | 1 | 0 | 1 | 0 | 0 | 0 | 2 |
| New Hampshire | 1 | 0 | 1 | 0 | 0 | 0 | 0 | 0 | 0 | 1 |
| New Jersey | 4 | 9 | 13 | 1 | 0 | 1 | 0 | 0 | 0 | 14 |
| New Mexico | 1 | 0 | 1 | 0 | 0 | 0 | 0 | 0 | 0 | 1 |
| New York | 13 | 3 | 16 | 2 | 0 | 2 | 0 | 0 | 0 | 18 |
| North Carolina | 28 | 5 | 33 | 0 | 0 | 0 | 1 | 1 | 2 | 35 |
| North Dakota | 0 | 0 | 0 | 0 | 0 | 0 | 0 | 0 | 0 | 0 |
| Ohio | 4 | 0 | 4 | 0 | 0 | 0 | 0 | 0 | 0 | 4 |
| Oklahoma | 4 | 7 | 11 | 3 | 0 | 3 | 0 | 1 | 1 | 15 |
| Oregon | 0 | 1 | 1 | 0 | 0 | 0 | 0 | 0 | 0 | 1 |
| Pennsylvania | 2 | 6 | 8 | 0 | 0 | 0 | 0 | 0 | 0 | 8 |
| Rhode Island | 2 | 0 | 2 | 0 | 0 | 0 | 0 | 0 | 0 | 2 |
| South Carolina | 4 | 4 | 8 | 0 | 0 | 0 | 0 | 0 | 0 | 8 |
| South Dakota | 0 | 0 | 0 | 0 | 0 | 0 | 0 | 0 | 0 | 0 |
| Tennessee | 15 | 22 | 37 | 4 | 0 | 4 | 2 | 0 | 2 | 43 |
| Texas | 22 | 31 | 53 | 0 | 0 | 0 | 0 | 0 | 0 | 53 |
| Utah | 0 | 1 | 1 | 0 | 0 | 0 | 0 | 0 | 0 | 1 |
| Vermont | 2 | 0 | 2 | 0 | 0 | 0 | 0 | 0 | 2 | 4 |
| Virginia | 17 | 29 | 46 | 1 | 0 | 1 | 2 | 0 | 2 | 49 |
| Washington | 13 | 6 | 19 | 0 | 0 | 0 | 0 | 0 | 0 | 19 |
| West Virginia | 0 | 0 | 0 | 0 | 0 | 0 | 0 | 1 | 1 | 1 |
| Wisconsin | 3 | 1 | 4 | 2 | 1 | 3 | 1 | 1 | 2 | 9 |
| Wyoming | 0 | 2 | 2 | 0 | 0 | 0 | 0 | 0 | 0 | 2 |
| Total | 423 | 331 | 754 | 39 | 5 | 44 | 17 | 12 | 29 | 827 |

[a]Includes cases that could not be ascribed to a specific ehrlichial agent because of antibody cross-reactivity.
[b]Probable case (single antibody titer of ≥ 64 by IFA).
[c]Confirmed case (fourfold change in antibody titer in paired serum samples by IFA).
